# Supplementary material for: A Simple Model-Based Approach to Inferring and Visualizing Cancer Mutation Signatures
Source: PLoS Genet. 2015 Dec 2;11(12):e1005657. doi: 10.1371/journal.pgen.1005657 (PMC4667891; doi:10.1371/journal.pgen.1005657)
Supplement: S1 Text — (PDF) [file pgen.1005657.s001.pdf]

Experiments on synthetic data

We begin by illustrating the ability of our method to extract "true" mutation signatures on simulated data. In these simulations a mutation pattern is defined by a substitution and the ± 2 flanking bases. We generated a set of mutations changing the number of cancer genomes ($I = 10, 25, 50, 100$), and the number of mutations for each cancer genome ($J = 10, 25, 50, 100, 250, 500, 1000$). The number of mutation signatures were set to $K = 5$ including background mutation ratio. The mutation feature parameters and membership parameters were generated by Dirichlet distribution,

$$\boldsymbol{f}_{k,l} \sim \mathrm{Dir}(\alpha \mathbf{1}),\ k = 1, \cdots, K,\ l = 1, \cdots, L. \tag{1}$$

$$\boldsymbol{q}_{i,k} \sim \mathrm{Dir}(\gamma \mathbf{1}),\ i = 1, \cdots, I, \tag{2}$$

where α and γ control the amount of dispersion for the mutation signature parameters and membership parameters, respectively. For example, when γ is small, most samples will have most of their mutations coming from a single signature (but not the same signature for each sample). When γ is large most samples will have mutations coming from all signatures in roughly equal proportions.

As Fig. 1(a) shows, we estimate the mutation signatures very accurately overall (see Fig. 1(b) for an example). As expected, accuracy improves as we increase the number of cancer genomes or mutations. Also, as the mutation feature dispersion decreases (α increases), accuracy decreases. This is expected: as α increases the signatures will become more "fuzzy", with probability mass spread over a large number of mutation patterns, and so harder to infer (In other words, larger α corresponds to a less informative motif). In contrast, accuracy was relatively insensitive to individual membership dispersion (γ), and estimates remain accurate even when most individuals have mutations in many different signatures.

In most cases, the log-likelihood stopped increasing at $K = 5$ mutation signatures, whereas the standard error of the estimated parameters started increasing past $K = 5$ (see Fig. 1(c)). We conclude that examining the trade-off between likelihood and standard-errors may be a helpful guide to selecting an appropriate number of mutation signatures.

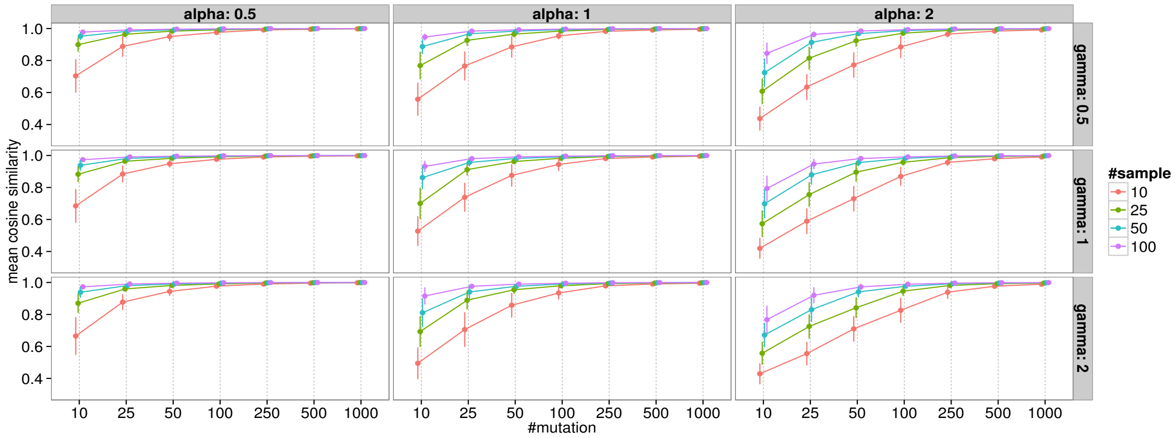

(a) The accuracy of the proposed approach for the simulated data when changing the number of samples, mutations, and the amounts of dispersion parameters (α and γ) for the mutation features and signature distribution parameters.

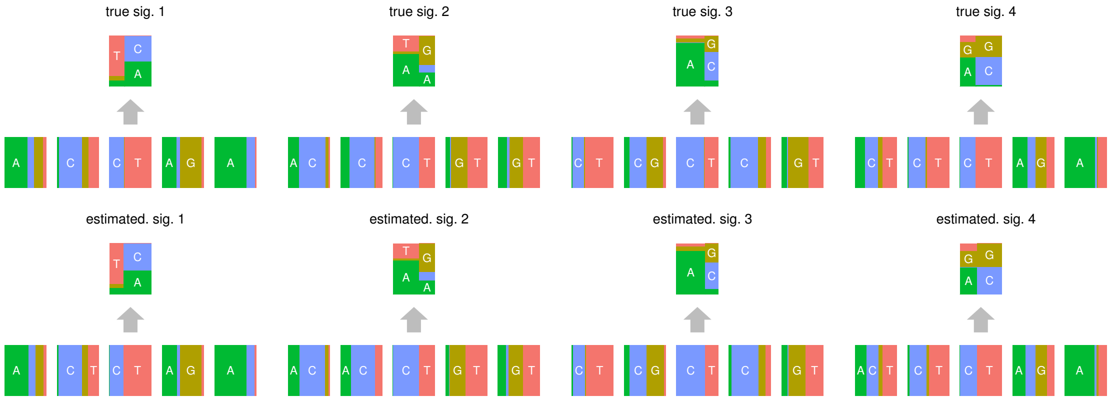

(b) An example of the relationship between true and estimated mutation signatures in the simulation. In this example, the numbers of cancer genomes and mutations for each cancer genome are 25 and 100, respectively, and the parameters α and γ are both set to 1. From this figure, we can see that fairly accurate estimation is possible even with moderate numbers of cancer genomes and mutations

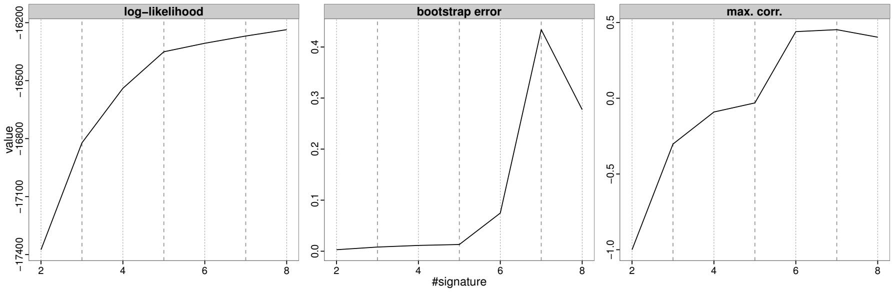

(c) The log-likelihood, bootstrap-errors and maximum correlation values among estimated membership parameters for several numbers of signatures K in the trial of the above figure.

Figure 1: The summary of simulation study.
