# Supplementary material for: A Simple Model-Based Approach to Inferring and Visualizing Cancer Mutation Signatures
Source: PLoS Genet. 2015 Dec 2;11(12):e1005657. doi: 10.1371/journal.pgen.1005657 (PMC4667891; doi:10.1371/journal.pgen.1005657)
Supplement: S2 Text — (PDF) [file pgen.1005657.s002.pdf]

Experiment on UCUT data with the various number of mutation signatures

We analyzed the data using our new model, with increasing number of mutation signatures $K = 2, 3, \ldots$. Results are shown in Figure 1. As expected, the likelihood increased with K. Bootstrap-errors started to increase at $K = 5$ (Figure 2).

With $K = 2$ (Figure 1(a)) we observed a mutation signature that appears to correspond to AA (T $>$ A substitutions at CpTpG sites with strong transcription strand specificity). Increasing to $K = 3$ introduced an additional mutation signature corresponding to the APOBEC enzyme (C $>$ [AGT] at TpCpN sites) (Figure 1(b)). Increasing to $K = 4$ introduced an additional signature (T $>$ A at NpTpN sites with strong strand specificity) that is somewhat similar to the AA signature (Figure 1(c)). The membership in the AA signature and this "AA-like" signature were correlated ($R = 0.77$; Figure 3). This additional signature may be just making up for the residual of the AA signature which the original AA signature could not explain due to a slight deviance of the probabilistic model.

The strong correlation among estimated membership parameters started to be shown at $K = 4$. Considering all these factors together, $K = 3$ seems to be a reasonable choice in terms of the interpretability, and we adopted $K = 3$.

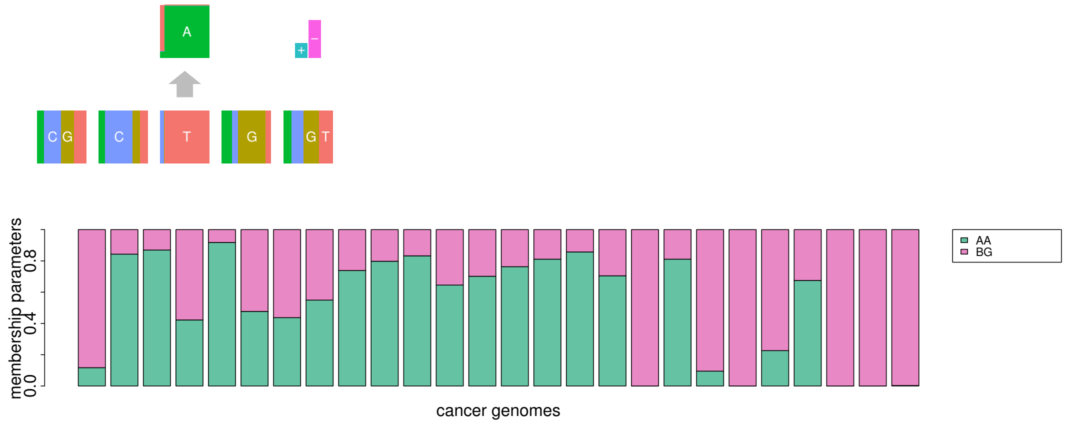

(a) $K = 2$

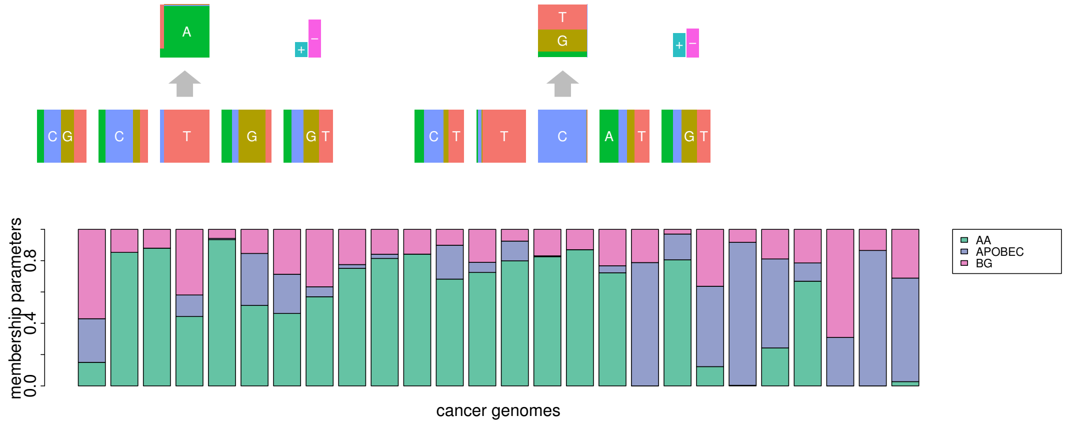

(b) $K = 3$

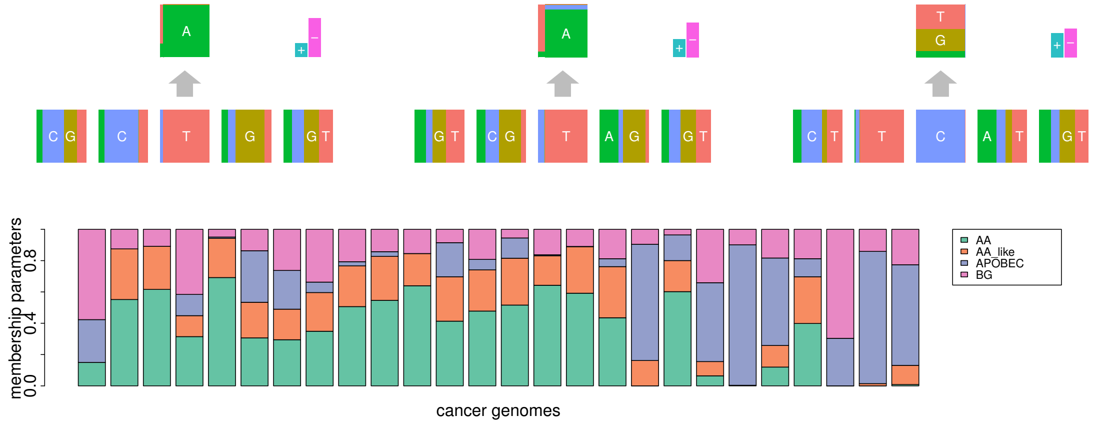

(c) $K = 4$

Figure 1: The result of estimated mutation signatures and membership parameters for UTUC data when changing the number of mutation signatures K.

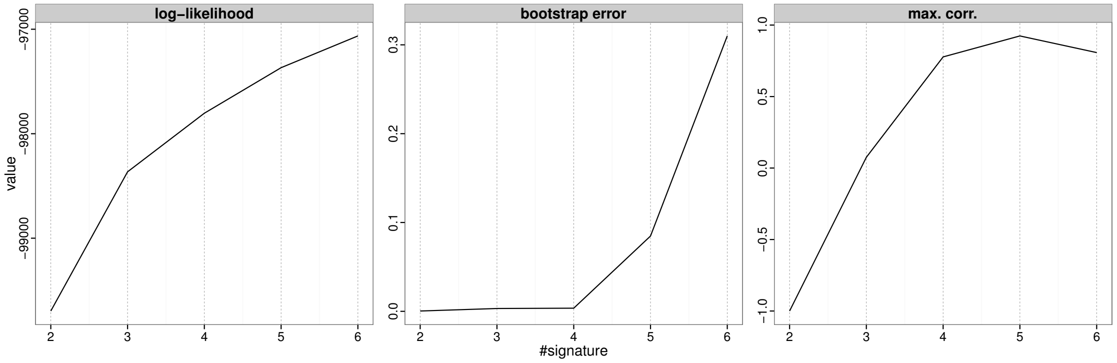

Figure 2: The log-likelihood, bootstrap-errors and maximum correlation values among estimated membership parameters for several numbers of signatures K in UTUC data.

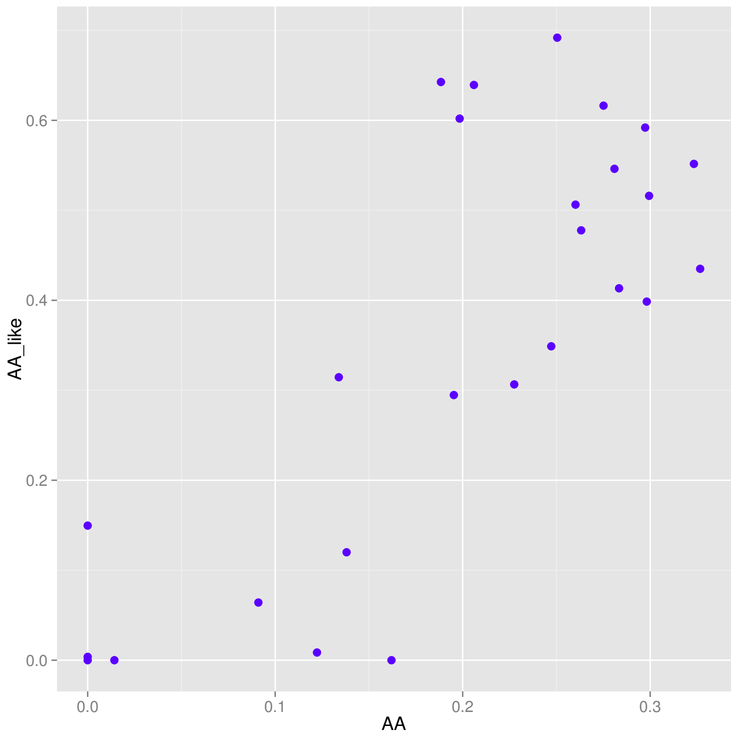

Figure 3: The relationships between the two membership parameters, AA (the first signature in the Figure 1(c)) and AA_like (the second signature in the Figure 1(c)) signatures.
