# Supplementary material for: A Simple Model-Based Approach to Inferring and Visualizing Cancer Mutation Signatures
Source: PLoS Genet. 2015 Dec 2;11(12):e1005657. doi: 10.1371/journal.pgen.1005657 (PMC4667891; doi:10.1371/journal.pgen.1005657)
Supplement: S3 Text — (PDF) [file pgen.1005657.s003.pdf]

Derivation of EM algorithm

The complete log-likelihood including missing data $\{\boldsymbol{z}_i\}$ for the proposed model is

$$\sum_{i=1}^{I}\sum_{j=1}^{J}\sum_{k=1}^{K} I(z_{i,j}=k)\Big(\sum_{l=1}^{L}\log f_{k,l,x_{i,j,l}} + \log q_{i,k}\Big).$$

Here, we introduce the variable for conditional probability for $z_{i,j}$ given the parameters and the mutation features $\boldsymbol{x}_{i,j}$,

$$\theta_{i,k,\boldsymbol{m}} = \Pr\big(z_{i,j}=k \big| \boldsymbol{x}_{i,j}=\boldsymbol{m}, \{\boldsymbol{f}_{k,l}\}, \{\boldsymbol{q}_i\}\big)$$

Note that this conditional probability just depends on the value of mutation feature $\boldsymbol{m}=(m_1,\cdots,m_L)$, not on the index j. Then, the expected complete log-likelihood augmented by Lagrange multipliers is calculated as

$$\sum_{i=1}^{I}\sum_{\boldsymbol{m}} g_{i,\boldsymbol{m}}\sum_{k=1}^{K}\theta_{i,k,\boldsymbol{m}}\Big(\sum_{l=1}^{L}\log f_{k,l,m_l} + \log q_{i,k}\Big) + \sum_{k=1}^{K}\sum_{l=1}^{L}\tau_{k,l}\big(1-\sum_{p=1}^{M_l} f_{k,l,p}\big) + \sum_{i=1}^{I}\rho_i\big(1-\sum_{k=1}^{K} q_{i,k}\big).$$

Differentiating it leads to following stationary equations:

$$\sum_{i=1}^{I}\sum_{\boldsymbol{m}:m_l=p} g_{i,\boldsymbol{m}}\theta_{i,k,\boldsymbol{m}} - \tau_{k,l}f_{k,l,p} = 0,\ (p=1,\cdots,M_l, k=1,\cdots,K,\ l=1,\cdots,L).,$$

$$\sum_{\boldsymbol{m}} g_{i,\boldsymbol{m}}\theta_{i,k,\boldsymbol{m}} - \rho_i q_{i,k} = 0,\ (k=1,\cdots,K, i=1,\cdots,I).$$

Then, by eliminating Lagrange multipliers, updating rules can be obtained.
