# Supplementary material for: A Simple Model-Based Approach to Inferring and Visualizing Cancer Mutation Signatures
Source: PLoS Genet. 2015 Dec 2;11(12):e1005657. doi: 10.1371/journal.pgen.1005657 (PMC4667891; doi:10.1371/journal.pgen.1005657)
Supplement: S4 Text — (PDF) [file pgen.1005657.s004.pdf]

Relationship with nonnegative matrix factorization

First, for ease of explanation, let assume that the full representation" representation ($L = 1$) is used. Suppose that each $\boldsymbol{m}$ has unique appropriate index from 1 to $|\boldsymbol{M}| = \prod_{l=1}^{L} M_l$ (the number of possible mutation patterns), so that $\boldsymbol{m}$ can be indices of matrices.

Let $G = \{g_{i,\boldsymbol{m}}\}$ denote the $I \times |\boldsymbol{M}|$ matrix, where $g_{i,\boldsymbol{m}}$ is the number of mutations whose mutation patters are equal to $\boldsymbol{m}$ in the i-th cancer genome. Nonnegative matrix factorization aims to find low rank decomposition, $G \sim \tilde{Q}F$, where $\tilde{Q} = \{\tilde{q}_{i,k}\}$ and $F = \{f_{k,\boldsymbol{m}}\}$ are nonnegative matrix, and row vectors of F are often restricted to be sum to one. We used the notation $\tilde{Q}$ instead of Q to represent that the row vectors of $\tilde{Q}$ are not normalized to sum to one in general.

For solving NMF, the previous study (Lee et al. 2000) used the following updating rule:

$$f_{k,m} \leftarrow f_{k,m} \frac{(\tilde{Q}^T G)_{k,m}}{(\tilde{Q}^T \tilde{Q} F)_{k,m}}, \quad \tilde{q}_{i,k} \leftarrow \tilde{q}_{i,k} \frac{(GF^T)_{i,k}}{(\tilde{Q}FF^T)_{i,k}},$$

that reduces the *Euclidean distance* $||G - \tilde{Q}F||$. Therefore, the optimization problem for the existing approach is

$$\begin{aligned} \text{minimize} \quad & ||G - \tilde{Q}F|| \\ \text{subject to} \quad & \sum_{\boldsymbol{m}} f_{k,\boldsymbol{m}} = 1, \ k = 1, \cdots, K \\ & f_{k,\boldsymbol{m}} \geq 0, \ k = 1, \cdots, K, \ \boldsymbol{m} \in M \\ & \tilde{q}_{i,k} \geq 0, \ i = 1, \cdots, I, \ k = 1, \cdots, K. \end{aligned} \tag{1}$$

On the other hand, there is another type of updating rule:

$$f_{k,m} \leftarrow f_{k,m} \frac{\sum_i \tilde{q}_{i,k} g_{i,m} / (\tilde{Q}F)_{i,m}}{\sum_i \tilde{q}_{i,k}},$$

$$\tilde{q}_{i,k} \leftarrow \tilde{q}_{i,k} \frac{\sum_m f_{k,m} g_{i,m} / (\tilde{Q}F)_{i,m}}{\sum_m f_{k,m}}.$$

that reduces the Kullback-Liebler Divergence:

$$KL(G||\tilde{Q}F) = \sum_{i,m} \Big(g_{i,m} \log \frac{g_{i,m}}{(\tilde{Q}F)_{i,m}} - g_{i,m} + (\tilde{Q}f)_{i,m} \Big).$$

In general cases including the independent representation, there is restrictions $f_{k,\boldsymbol{m}} = \prod_l f_{k,l,m_l}$ by smaller set of parameters. Let us consider the following optimization problem with the Kullback-Liebler Divergence and the restrictions on F:

$$\begin{aligned} \text{minimize} \quad & KL(G||\tilde{Q}F) \\ \text{subject to} \quad & f_{k,\boldsymbol{m}} = \prod_l f_{k,l,m_l}, \ k = 1, \cdots, K, \ \boldsymbol{m} \in M \\ & f_{k,l,p} \geq 0, \ k = 1, \cdots, K, \ \boldsymbol{m} \in M \\ & \tilde{q}_{i,k} \geq 0, \ i = 1, \cdots, I, \ k = 1, \cdots, K. \end{aligned} \tag{2}$$

In fact, this is equivalent to the proposed method, whose optimization problem can be written as:

$$\begin{aligned}
\text{maximize} \quad & L(Q,F|G)\big(=\sum_{i,m} g_{i,m}\log(QF)_{i,m}\big) \\
\text{subject to} \quad & f_{k,\boldsymbol{m}} = \prod_{l} f_{k,l,m_l},\ k=1,\cdots,K,\ \boldsymbol{m}\in M \\
& f_{k,l,p} \geq 0,\ k=1,\cdots,K,\ \boldsymbol{m}\in M \\
& \sum_{k} q_{i,k} = 1,\ i=1,\cdots,I \\
& q_{i,k} \geq 0,\ i=1,\cdots,I,\ k=1,\cdots,K.
\end{aligned} \tag{3}$$

Proposition 1 *When $(Q,F)=(Q^*,F^*)$ is an optimal solution of the optimization problem (3), then $(\tilde{Q},F)=(R^*Q^*,F^*)$ is an optimal solution of the optimization problem (2). On the other hand, when $(\tilde{Q},F)=(\tilde{Q}^*,F^*)$ is an optimal solution of the optimization problem (2), then $(Q,F)=(R^{*-1}\tilde{Q}^*,F^*)$ is an optimal solution of the optimization problem (3), where $R^*=diag(r_1^*,\cdots,r_I^*), r_i^*=\sum_{\boldsymbol{m}} g_{i,\boldsymbol{m}}, i=1,\cdots,I$.*

Proof. This is because

$$KL(G||\tilde{Q}F) = -\sum_i \Big((\sum_m g_{i,m})\log\tilde{r}_i - \tilde{r}_i \Big) - L(Q,F|G) + (\text{constant value}),$$

where Q is row-normalized matrix for $\tilde{Q}$, $\tilde{r}_i=\sum_k q_{i,k}$ for each i, and $(\sum_m g_{i,m})\log\tilde{r}_i - \tilde{r}_i$ takes its maximum at $\tilde{r}_i = r_i^*$. □
