# Supplementary material for: A Simple Model-Based Approach to Inferring and Visualizing Cancer Mutation Signatures
Source: PLoS Genet. 2015 Dec 2;11(12):e1005657. doi: 10.1371/journal.pgen.1005657 (PMC4667891; doi:10.1371/journal.pgen.1005657)
Supplement: S1 Table — In the independent representation, the elements of vector show substitution patterns, 5′ adjacent bases and 3′ adjacent bases, respectively. For substitution pattens, 1 to 6 values are assigned to C>A, C>G, C>T, T>A, T>C and T>G in this order. For 5′ and 3′ adjacent bases, 1 to 4 values are assigned to A, C, G and T. Note that the original base is fixed to C or T to remove the redundancy of complement sequences. (PDF) [file pgen.1005657.s005.pdf]

Supplementary Table 1

mutation pattern	full model	independent model
L	1	3
$\boldsymbol{M}$	(96)	(6, 4, 4)
ApCpA → ApCpA	(1)	(1, 1, 1)
ApCpC → ApApC	(2)	(1, 1, 2)
ApCpG → ApApG	(3)	(1, 1, 3)
ApCpT → ApApT	(4)	(1, 1, 4)
CpCpA → CpApA	(5)	(1, 2, 1)
...	...	...
ApCpA → ApGpA	(17)	(2, 1, 1)
...	...	...
TpTpT → TpGpT	(96)	(6, 4, 4)
