# Supplementary material for: A Simple Model-Based Approach to Inferring and Visualizing Cancer Mutation Signatures
Source: PLoS Genet. 2015 Dec 2;11(12):e1005657. doi: 10.1371/journal.pgen.1005657 (PMC4667891; doi:10.1371/journal.pgen.1005657)
Supplement: S2 Table — (PDF) [file pgen.1005657.s006.pdf]

Supplementary Table 2

cancer type	# mutation signature
AML	2
ALL	3
Bladder	3
Breast	4
Cervix	3
CLL	3
Colorectum	5
Esophageal	4
Glioblastoma	3
Glioma-Low-Grade	3
Head-and-Neck	5
Kidney-Chromophobe	3
Kidney-Clear-Cell	3
Kidney-Papillary	3
Liver	3
Lung-Adeno	4
Lung-Small-Cell	4
Lung-Squamous	3
Lymphoma-B-Cell	4
Medulloblastoma	3
Melanoma	4
Myeloma	3
Neuroblastoma	3
Ovary	3
Pancreas	4
Pilocytic-Astrocytoma	4
Prostate	2
Stomach	5
Thyroid	4
Uterus	4
